# Splicing Analysis of 16 *PALB2* ClinVar Variants by Minigene Assays: Identification of Six Likely Pathogenic Variants

**DOI:** 10.3390/cancers14184541

**Published:** 2022-09-19

**Authors:** Alberto Valenzuela-Palomo, Lara Sanoguera-Miralles, Elena Bueno-Martínez, Ada Esteban-Sánchez, Inés Llinares-Burguet, Alicia García-Álvarez, Pedro Pérez-Segura, Susana Gómez-Barrero, Miguel de la Hoya, Eladio A. Velasco-Sampedro

**Affiliations:** 1 Splicing and Genetic Susceptibility to Cancer, Unidad de Excelencia Instituto de Biología y Genética Molecular, Consejo Superior de Investigaciones Científicas (CSIC-UVa), 47003 Valladolid, Spain; 2 Molecular Oncology Laboratory, Hospital Clínico San Carlos, IdISSC (Instituto de Investigación Sanitaria del Hospital Clínico San Carlos), 28040 Madrid, Spain; 3 Facultad de Ciencias de la Salud, Universidad Alfonso X “El Sabio”, Avda. de la Universidad 1, Villanueva de la Cañada, 28691 Madrid, Spain

**Keywords:** hereditary breast cancer, cancer susceptibility genes, *PALB2*, aberrant splicing, functional assay, minigenes, clinical interpretation

## Abstract

**Simple Summary:**

*PALB2* pathogenic variants confer high risk of breast cancer. Here, we have analyzed the impact of *PALB2* variants on splicing, a gene expression step that removes introns to form the mature messenger RNA. This process is performed by the splicing machinery through the recognition of specific sequences, namely the 3′ and 5′ splice sites, which determine the exon ends. Variants at these sequences may trigger anomalous splicing and aberrant transcripts that may be associated with a disease. To test the impact of variants on splicing, we used a biotechnological tool called minigene, which replicates, at small-scale, the human gene of interest. Thus, we checked 16 *PALB2* variants at the intron/exon boundaries using the minigene mgPALB2_ex1-3. We found that twelve variants disrupted splicing, six of which could be classified as likely pathogenic. These results facilitate the clinical management of carrier patients and families since they may benefit from tailored prevention protocols and therapies.

**Abstract:**

*PALB2* loss-of-function variants are associated with significant increased risk of breast cancer as well as other types of tumors. Likewise, splicing disruptions are a common mechanism of disease susceptibility. Indeed, we previously showed, by minigene assays, that 35 out of 42 *PALB2* variants impaired splicing. Taking advantage of one of these constructs (mgPALB2_ex1-3), we proceeded to analyze other variants at exons 1 to 3 reported at the ClinVar database. Thirty-one variants were bioinformatically analyzed with MaxEntScan and SpliceAI. Then, 16 variants were selected for subsequent RNA assays. We identified a total of 12 spliceogenic variants, 11 of which did not produce any trace of the expected minigene full-length transcript. Interestingly, variant c.49-1G > A mimicked previous outcomes in patient RNA (transcript ∆(E2p6)), supporting the reproducibility of the minigene approach. A total of eight variant-induced transcripts were characterized, three of which (∆(E1q17), ∆(E3p11), and ∆(E3)) were predicted to introduce a premature termination codon and to undergo nonsense-mediated decay, and five (▼(E1q9), ∆(E2p6), ∆(E2), ▼(E3q48)-a, and ▼(E3q48)-b) maintained the reading frame. According to an ACMG/AMP (American College of Medical Genetics and Genomics/Association for Molecular Pathology)-based classification scheme, which integrates mgPALB2 data, six *PALB2* variants were classified as pathogenic/likely pathogenic, five as VUS, and five as likely benign. Furthermore, five ±1,2 variants were catalogued as VUS because they produced significant proportions of in-frame transcripts of unknown impact on protein function.

## 1. Introduction

Hereditary breast cancer (BC) is a highly heterogenous genetic disease, in which more than 20 genes of the DNA repair pathway have been proposed as breast cancer susceptibility genes [1]. Historically, genetic testing was focused on the main BC genes *BRCA1* and *BRCA2* by different methods [2,3]. The development of next-generation sequencing (NGS) enabled the development of panels of cancer predisposing genes and the simultaneous sequencing of multiple genes, thus boosting efficiency and cost-effectiveness. Recently, two large-scale sequencing studies, which sequenced a panel of breast cancer genes in more than 170,000 women, refined the BC/OC genetic predisposition spectrum [4,5]. At least eight genes were found to be significantly associated with breast cancer risk: *BRCA1* (MIM#113705), *BRCA2* (MIM#600185), *ATM* (MIM#607585), *BARD1* (MIM #601593), *CHEK2* (MIM#604373), *PALB2* (MIM#610355), *RAD51C* (MIM#602774), and *RAD51D* (MIM#602954) [4,5,6]. Biallelic loss-of-function variants of *PALB2* (also known as *FANCN*) and other BC susceptibility genes, such as *BRCA2*, *RAD51C*, and *BRCA1*, cause Fanconi anemia [7], which is characterized by a high genomic instability and increased cancer predisposition.

The partner and localizer of BRCA2 (PALB2) interacts with BRCA1 and BRCA2 and is implicated in repair of double-strand DNA breaks by homologous recombination. While BRCA1 recruits PALB2 at the sites of DNA damage, PALB2 stabilizes BRCA2 during formation of the RAD51 nucleoprotein filament [8,9]. *PALB2* loss-of-function variants confer high risk of developing breast cancer (BC) as well as other types of cancers [10,11,12]. Two recent reports have shown that *PALB2* protein truncating variants are associated with a significantly increased risk of breast cancer (BC relative risk 3.83 and 5.02, respectively) and accounts for 9.5–10.1% of the protein truncating variants of the eight core BC genes mentioned above (0.39–0.56% of all BC cases) [4,5]. Furthermore, associations with estrogen-negative and triple-negative BC are even higher, with relative risks of 7.35 (4.25–12.72) and 10.36 (6.42–16.71), respectively [4]. Hence, *PALB2* belongs to the high-risk category of BC susceptibility genes together with *BRCA1* and *BRCA2*.

On the other hand, a deleterious effect on gene function cannot be assigned for a relevant proportion of variants detected in patients, the so-called variants of uncertain clinical significance (VUS) [13]. In the case of *PALB2*, VUS frequency is approximately four times greater than that of pathogenic variants [5]. Consequently, they represent a challenge in genetic counselling because cancer risk assessment in VUS carriers is only based on cancer family history [14]. Apart from protein translation, there are other gene expression steps that may be targeted by disease-causing variants, such as transcription, splicing, as well as other post-transcriptional mechanisms [15,16,17,18]. Functional studies of these processes provide key information for the clinical interpretation of VUS.

The splicing process Is controlled by a large collection of splicing factors and cis-acting sequences that include: the 5′ or donor (GT) and the 3′ or acceptor (AG) splice sites (5′SS and 3′SS, respectively), the polypyrimidine tract and the branch point, as well as exonic and intronic elements that promote (enhancers) or repress (silencers) exon recognition [19]. All these motifs may be targets of splicing-disrupting mutations (spliceogenic variants) so that an unexpectedly large fraction of variants can actually induce splicing anomalies [17,20,21]. In fact, it was estimated that about 62% of pathogenic variants impair splicing [22]. Interestingly, several cancer susceptibility genes, such as *MLH1*, *MSH2*, and *PMS2*, are enriched in spliceogenic variants [23].

We have focused our efforts on the study of the impact of genetic variants on the splicing of the BC genes by minigene assays, by which we found a large proportion of spliceogenic variants [24]. We comprehensively analyzed by minigene assays several BC susceptibility genes, such as *BRCA2* [25], along with *RAD51C, RAD51D, PALB2*, and *ATM* within the framework of the European Project BRIDGES (Breast Cancer Risk after Diagnostic Gene Sequencing; https://bridges-research.eu/, accessed on 12 July 2022) [26,27,28,29].

In a recent work, we studied the *PALB2* gene and tested, in three different minigenes, 42 candidate BRIDGES variants [28], 35 of which disrupted splicing, with 23 of them being classified as pathogenic or likely pathogenic, demonstrating the usefulness of minigenes for RNA assays and clinical interpretation of variants. Moreover, these constructs are highly valuable since any other potentially spliceogenic variants of the gene of interest can be so assayed.

Taking advantage of the minigene mgPALB2_ex1-3, we selected 31 ClinVar splice-site variants located at exons 1–3 (https://www.ncbi.nlm.nih.gov/clinvar/, accessed on 3 August 2021) to carry out splicing assays of the potentially damaging variants.

## 2. Materials and Methods

### 2.1. Variant and Transcript Annotations

The analysis of the ClinVar data identified a total of 31 variants at exons 1, 2, and 3 and flanking intronic sequences located at the *PALB2* 5′ and 3′ splice sites (5′SS and 3′ SS, respectively), defined for the purpose of the present study as: (i) intron/exon (IVS –10 to –1/2 nt) boundaries (3′SS) and (ii) exon/intron (2 nt/IVS +1 to +6) boundaries (5′SS). Variants, transcripts and predicted protein products were described according to the Human Genome Variation Society (HGVS) guidelines (https://varnomen.hgvs.org/, accessed on 1 June 2022), using the Ensembl reference transcript ID ENSG00000083093 (Genbank NM_024675.4)**.** We also annotated splicing events according to a former shortened description [30,31].

### 2.2. Bioinformatics: Databases and In Silico Studies

All the 31 *PALB2* ClinVar splice-site variants at exons 1, 2, and 3 were analyzed with MaxEntScan (MES) http://hollywood.mit.edu/burgelab/maxent/Xmaxentscan_scoreseq.html, accessed on 3 August 2021) to identify potentially spliceogenic variants [32]. Candidate variants were analyzed under the following criteria [28]: (i) splice site disruption at the ±1,2 (AG/GT) positions and (ii) relevant MES score changes (≥15%) [33,34]. All variants were further evaluated with the splice-site predictor SpliceAI (https://spliceailookup.broadinstitute.org/, accessed on 3 August 2021) [35]. SpliceAI outputs were helpful to predict putative splicing outcomes based on a “two score” approach (e.g., donor loss + acceptor loss predicts exon skipping, while donor loss + donor gain predicts a donor shift). SpliceAI parameters were as follows: genome version hg38; score type raw; max distance 10,000 nt; Illumina’s pre-computed scores yes. Scores range 0–1 is interpreted as probability of impact on splicing with the following cutoffs: 0.2–0.49 (high recall), 0.5–0.79 (recommended), and >0.8 (high precision). SpliceAI was not herein used to filter out variants. On basis of the MES outcomes, we decided to carry out the subsequent splicing assays for 16 potentially spliceogenic variants.

### 2.3. Minigene Construction and Mutagenesis

**Figure 1:**
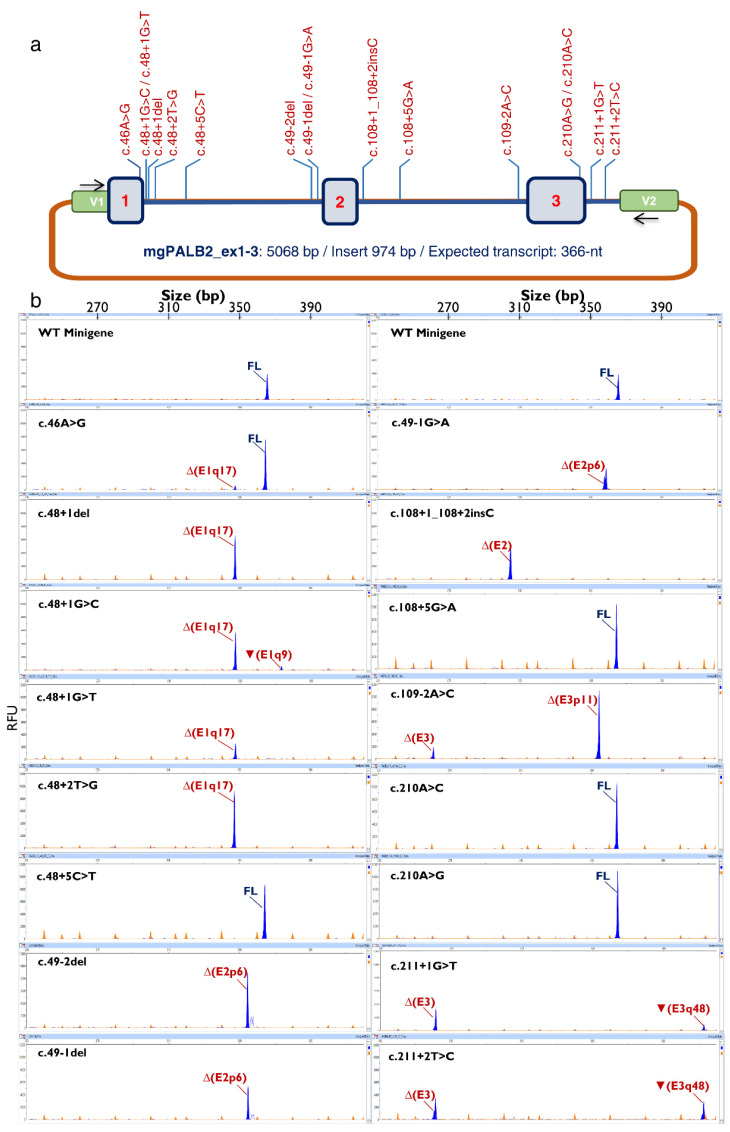
Minigene splicing assays of selected *PALB2* variants. (**a**) Map of variants in the minigene mgPALB2_ex1–3. V1 and V2 are the vector exons while variants are shown in red above the minigene construct (**b**) Fluorescent fragment analysis of 16 variants. The electropherogram of the wild-type minigene is shown on the top of each column. FAM-labelled products (blue peaks) were run together with LIZ-600 (orange peaks) as size standard (FL, minigene full-length transcript). The x-axis indicates size in bp (electropherograms on the top) and the y-axis represents relative fluorescence units (RFU).

The minigene mgPALB2_ex1–3 was built in the splicing vector pSAD as previously reported [28,36,37] (Figure 1a). In brief, this construct contains a 974 bp insert (final minigene size: 5068 bp) that includes exons 1 to 3. This construct has the special feature of a chimeric exon 1 composed of vector exon 1 and *PALB2* exon 1 so that 5′SS variants of exon 1 can be tested [28].

The wild-type minigene was used as template to generate 16 DNA ClinVar variants by site-directed mutagenesis with the QuikChange Lightning Kit (Agilent, Santa Clara, CA, USA) (Table 1). All mutant constructs were confirmed by sequencing (Macrogen, Madrid, Spain).

### 2.4. Splicing Functional Assays

Approximately 2 × 10^5^ MCF-7 and MDA-MB-231 cells were seeded in four-well plates (Nunc, Roskilde, Denmark) to grow up to 90% confluency in 0.5 mL of medium (MEME, 10% fetal bovine serum, 2 mM glutamine, 1% non-essential amino acids, and 1% penicillin/streptomycin). Then, using a standard protocol of transfection, MCF-7 cells were transfected with either the wild-type or the mutant minigenes. To inhibit nonsense-mediated decay (NMD), cells were incubated with cycloheximide 300 μg/mL (Sigma-Aldrich, St. Louis, MO) for 4 h. RNA was extracted after 48 h and purified with the Genematrix Universal RNA purification Kit (EURx, Gdansk, Poland) with on-column DNAse I digestion to degrade genomic DNA that could interfere in RT-PCR. Retrotranscription was carried out with specific primers of exons V1 and V2 of the pSAD^®^ vector as previously described [26,28,38]. The expected size of the minigene mgPALB2_ex1–3 full-length (mgFL) transcript was 366 nt. To estimate the relative abundance of all transcripts, semi-quantitative fluorescent RT-PCRs (26 cycles) were performed with pSPL3_RT-FW and FAM-RTpSAD-RV. FAM-labeled products were run with LIZ-600 Size Standards at the Macrogen facility and analyzed with the Peak Scanner software V1.0 (Life Technologies, Carlsbad, CA, USA). Three independent experiments for each variant were carried out to calculate the average relative proportions of each transcript and the corresponding standard deviations.

### 2.5. Clinical Classification of PALB2 Variants

We performed a tentative clinical classification of 16 *PALB2* variants according to ACMG/AMP-based guidelines. We used a Bayesian-ACMG/AMP point system that shows higher plasticity in combining different ACMG/AMP criteria and strengths of evidence [39,40]. Point-based variant classification categories are defined as follows: pathogenic (P) ≥ +10; likely pathogenic (LP) +6 to +9; variant of uncertain significance (VUS) 0 to +5; likely benign (LB) −1 to −6; and benign (B) ≤ −7. The mgPALB2 read-outs were included in the classification system as observable PVS1_O or BP7_O evidence codes of variable strength depending on the splicing outcome (P, supporting (±1 point); M, moderate (±2); S, strong (±4); VS, very strong (±8)) [29,41]. This score is deduced from the presumed impact of all the transcripts generated by a particular variant. To interpret variants producing ≥2 transcripts, we applied the following rules: (i) decode/separate mgPALB2-readouts into individual components (transcripts); (ii) apply ACMG/AMP-based evidence levels to each individual transcript; and (iii) deduce a global PVS1_O (or BP7_O) code strength based on the relative contribution of individual transcripts to the overall expression. Thus, if pathogenic (or benign) supporting transcripts contribute ≥90% to the overall expression level, PVS1_O (or BP7_O) codes are applied. If different transcripts support different pathogenic evidence strengths, the lowest strength contributing >10% to the overall expression is selected as overall evidence strength. At present, ≥90% and ≥10% cut-offs of the overall mgPALB2 expression are merely operational. Recently, we already used a similar approach to deal with those *PALB2/ATM/RAD51C* minigene readouts that yielded several transcripts per variant [28,29,41].

We considered that functional splicing data (PVS1_O/BP7_O) override predictive splicing codes PVS1 (GT-AG splice site variants) and PP3/BP4 (non-GT-AG variants) so that the latter does not contribute to our variant classification. Otherwise, internal inconsistencies would arise in the ACMG/AMP classification system (e.g., IVS + 1 and IVS + 5 variants with identical splicing impact would score very differently). Furthermore, the ACMG/AMP system implicitly assumes that each piece of evidence contributing to the final classification is independent, which is an assumption barely met by predictive and functional splicing codes, as most splicing analyses (including our mgPALB2 ones) are performed for bioinformatically pre-selected variants. These issues have been extensively discussed elsewhere [28,29,41]. The rarity code PM2 was considered with allele frequency ≤ 0.001% at gnomADv2.1.1 (https://gnomad.broadinstitute.org; accessed on 21 June 2022) decreasing, so PM2 evidence strength to “supporting” as previously reported [29]. For *PALB2* variants absent on gnomADv2.1.1, the number of interrogated alleles (allele number) was determined using data of the closest available SNP (≤5 nt apart from the variant of interest).

Since no specific *PALB2* recommendations exist for missense variants, we applied general recommendations recently published by ClinGen SVI [42]. Specifically, REVEL ≥0.8 supporting pathogenic (moderate strength), REVEL ≤ 0.4 supporting benign (moderate strength), and 0.4 < REVEL < 0.8 supporting neither pathogenic nor benign. To obtain REVEL scores, we ran the built-in Ensembl Variant Effect Predictor (www.ensembl.org/Tools/VEP; accessed on 1 June 2022).

## 3. Results and Discussion

### 3.1. Bioinformatics Analysis of ClinVar Variants

A total of 31 ClinVar variants comprising 66 submissions to the ClinVar database were chosen from the intron/exon boundaries of *PALB2* exons 1 to 3. All the 31 variants were bioinformatically analyzed with MaxEntScan, 16 of which met the criteria indicated in Materials and Methods, so they were selected for subsequent minigene RNA assays (Table 2). Twelve of these variants targeted splice donor sites, while the remaining four targeted acceptor sites. The 16 MES-selected variants were also analyzed by SpliceAI (Table 3). Changes c.48 + 5C > T and c.108 + 5G > A were not predicted to affect splicing.

### 3.2. Minigene Splicing Assays of Candidate Variants

**Figure 2:**
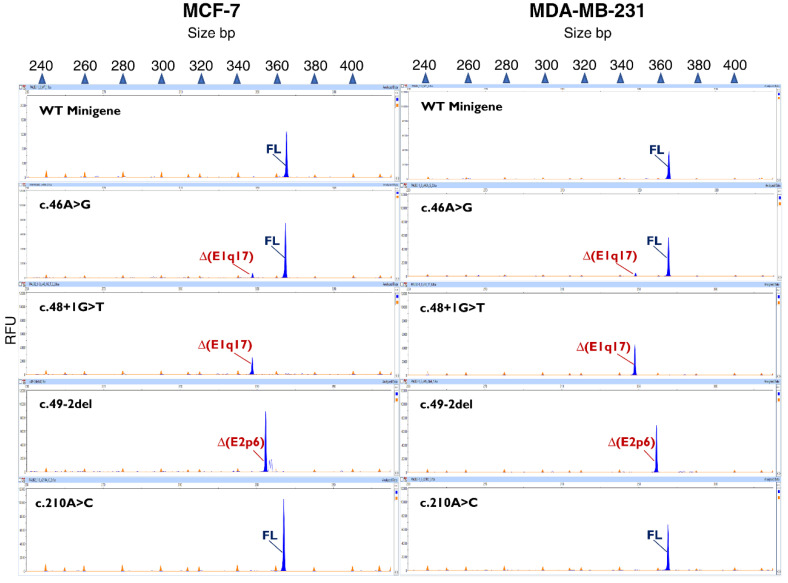
Reproducibility of PALB2 RNA assays in MDA-MB-231 (left) and MCF-7 (right) cells. The wild-type and mutant minigenes of c.46A > G, c.48 + 1G > T, c.49-2del, and c.210A > C were tested in MCF-7 and MDA-MB-231 cells. RT-PCR products were run by fluorescent fragment electrophoresis using LIZ-600 as size standard. The x-axis indicates size in bp (electropherograms on the top) and the y-axis represents relative fluorescence units (RFU).

The 16 variants were introduced into the wild-type minigene mgPALB2ex1–3 by site-directed mutagenesis and assayed in MCF-7 cells. Twelve variants impaired splicing, eleven of which showed a total impact, as the minigene full-length transcript was absent (Figure 1b, Table 4). These 11 variants affected the AG/GT (±1,2) dinucleotides of the 3′SS and 5′SS and showed the strongest impacts on MES scores (Table 2). In contrast, partial splicing anomalies were found in variants at other splice-site positions. Actually, we noticed weak or no splicing effects for those variants involving the antepenultimate nt of exon 1 (c.46A > G), +5 nt of introns 1 and 2 (c.48 + 5C > T and c.108 + 5G > A, respectively), and penultimate nt of exon 3 (c.210A > G and c.210A > C). Splicing disruptions of variants at positions other than ±1,2 are particularly difficult to predict, as we have pointed out in previous reports [28]. In this study, leaky variants (those that generate non-negligible levels of full-length transcripts) were associated with moderate reductions in the MES score (−19.3% to −29.8%, Table 2). To test the reproducibility of the minigene assay in different cell lines, four variants (c.46A > G, c.48 + 1G > T, c.49-2del, and c.210A > C) were also assayed in the triple-negative breast cancer cell line MDA-MB-231. All the variants mimicked the splicing patterns characterized in MCF-7 cells (Figure 2). Moreover, variant c.49-1G > A replicated the splicing outcomes formerly characterized in patient RNA [43], confirming the reproducibility of minigene assays (Table 3).

**Figure 3:**
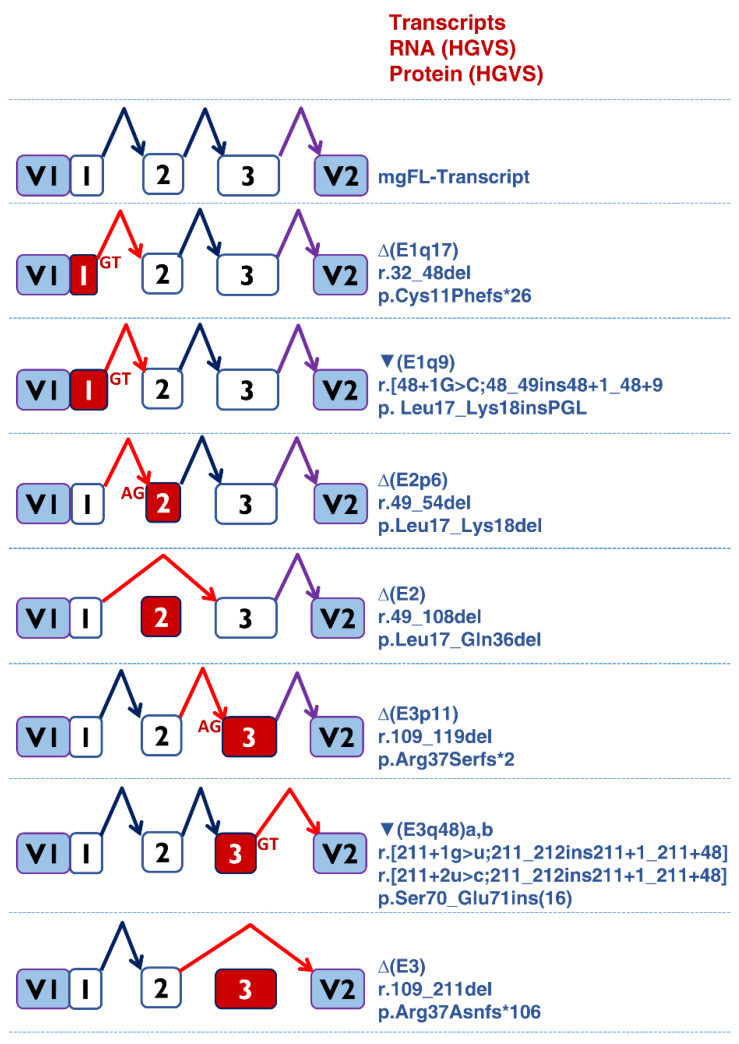
Transcripts produced by *PALB2* variants. Diagrams of the splicing reactions. Exons and the splicing reactions are indicated by boxes and elbow arrows, respectively. Anomalous events, exon skipping or alternative site usage (AG or GT sites) and exons are indicated in red. The impact of each transcript at the RNA and protein levels are described following the Human Genome Variation Society (HGVS) recommendations (right).

A total of eight different anomalous transcripts were characterized (Table 4, Figure 3). Three transcripts (Δ(E1q17), Δ(E3p11), Δ(E3)])are predicted to introduce a premature termination codon triggering the NMD surveillance mechanism (PTC-NMD) [28,44], while the remaining five isoforms, including two versions of ▼(E3q48) (a and b), maintained the reading frame. Minigene assays, together with fluorescent fragment analysis, displayed simplicity, robustness, high resolution, and sensitivity. This strategy allowed us to detect splicing alterations introducing small size changes (i.e., insertion of 9 nt or ▼(E1q9) or deletion of 6 nt, ∆(E2p6)) as well as some transcripts representing a minor contribution to the overall mgPALB2 expression (i.e., c.46A > G: ∆(E1q17), 7.5%; c.48 + 1G > C: ▼(E1q9), 9.2%; Table 4). 

**Table 2:** Bioinformatics analysis of *PALB2* variants with Max Ent Scan.

*PALB2* VARIANTS ^1^	# ClinVar Records ^2^	EXON/INTRON	MES wt	MES m ut	MES Score Change ^3^	Cryptic/De novo Splice Sites ^4^
c.46A > G	1	Exon 1	5.74	4.05	−29.4%	
c.48 + 1del	1	IVS1	5.74	−12.45	− 316.9%	
c.48 + 1G > C	3	IVS1	5.74	−2.53	− 144.1%	
c.48 + 1G > T	1	IVS1	5.74	−2.76	− 148.1%	
c.48 + 2T > G	1	IVS1	5.74	−1.9	− 133.1%	
c.48 + 5C > T	3	IVS1	5.74	4.05	−29.4%	
c.48 + 6G > C	1	IVS1	5.74	5.96	+3.8%	
c.49-2del	1	IVS1	9.28	1.16	−87.5%	3’SS: 5.47 26 nt upstream 3′SS: 8.65, 6 nt downstream
c.49-1del	1	IVS1	9.28	−7.59	−181.8%	3’SS: 5.47 26 nt upstream 3′SS: 8.76, 6 nt downstream
c.49-1G > A	1	IVS1	9.28	0.53	−94.3%	3’SS: 5.47 26 nt upstream 3′SS: 7.53, 6 nt downstream
c.50T > G	2	Exon 2	9.28	9.02	−2.8%	3’SS: 5.47 26 nt upstream
c.50dup	1	Exon 2	9.28	9.25	−0.3%	3’SS: 5.47 26 nt upstream
c.106C > T	4	Exon 2	10.86	9.66	−11%	de novo 5’SS: 3.81 4 nt downstream
c.108G > A	1	Exon 2	10.86	10.08	−7.2%	
c.108 + 1_108 + 2insC	1	IVS2	10.86	−4.16	−138.3%	
c.108 + 4A > G	3	IVS2	10.86	10.28	-5.3%	
c.108 + 5G > A	1	IVS2	10.86	8.76	−19.3%	
c.108 + 6T > C	1	IVS2	10.86	9.88	–9%	
c.109-2A > C	3	IVS2	10.06	2.02	−79.9%	3′SS: 5.47, 11 nt downstream
c.109C > G	2	Exon 3	10.06	11.86	+11.8%	
c.109C > T	5	Exon 3	10.06	9.82	−2.4%	
c.109C > A	7	Exon3	10.06	10.18	+1.2%	
c.110G > T	2	Exon 3	10.06	9.69	−3.7%	
c.110G > A	9	Exon 3	10.06	9.64	−4.2%	
c.111T > C	3	Exon 3	10.06	10.3	+2.4%	
c.210A > G	2	Exon 3	8.76	6.15	− 29.8%	5′SS: 7.88, 48 nt downstream
c.210A > C	1	Exon 3	8.76	6.89	− 21.3%	5′SS: 7.88, 48 nt downstream
c.211 + 1G > T	1	IVS3	8.76	0.26	–97.0%	5′SS: 7.88, 48 nt downstream
c.211 + 2T > C	1	IVS3	8.76	1.01	− 88.5%	5′SS: 7.88, 48 nt downstream
c.211 + 4A > G	1	IVS3	8.76	7.25	−17.2%	5′SS: 7.88, 48 nt downstream
c.211 + 6T > A	1	IVS3	8.76	8.59	−1.9%	5′SS: 7.88, 48 nt downstream

^1^ Selected variants are shown in red. ^2^ #, number of ClinVar Records; ^3^ MES score changes (∆%): mutant (mut) vs. wild type (wt). ^4^ Positions of cryptic/de novo splice sites are relative to the corresponding canonical splice site.

**Table 3:** SpliceAI predictions, mgPALB2 read-outs, and experimental splicing data in carriers.

*PALB2* Variants ^1^	SpliceAI ^2^						mgPALB2 Read-out (>10%)	Experimental Data in RNA from Carriers
	AL (>20%)	DL (>20%)	AG (>20%)	DG (>20%)	Predicted Splicing Outcome			
**c.46A > G**	-	-	-	0.27 (+11)	-		mgFL (92.5%); ∆(E1q17) (7.5%)	Normal [45]
c.48 + 1del	-	0.94 (−2)	-	0.25 (−171)	∆(E1q169)		∆(E1q17) (100%)	
c.48 + 1G > C	-	0.94 (−1)	-	0.45 (+8)	▼(E1q9)		∆(E1q17) (90.8%)/▼(E1q9) (9.2%)	
c.48 + 1G > T	-	0.94 (−1)	-	0.30 (+8)	▼(E1q9)		∆(E1q17) (100%)	
c.48 + 2 T > G	-	0.94 (−2)	-	-	-		∆(E1q17) (100%)	
**c.48 + 5C > T**	-	-	-	-	-		FL (100%)	
**c.49-2del**	0.98 (+1)	-	0.74 (+7)	-	∆(E2p6)		∆(E2p6) (100%)	
**c.49-1del**	0.98 (+1)	-	0.79 (+6)	-	∆(E2p6)		∆(E2p6) (100%)	
**c.49-1G > A**	0.98 (+1)	-	0.65 (+7)	-	∆(E2p6)		∆(E2p6) (100%)	∆(E2p6) [43]
**c.108 + 1_108 + 2insC**	0.96 (−61)	0.99 (−2)	-	-	∆(E2)		∆(E2) (100%)	
**c.108 + 5G > A**	-	-	-	-	-		FL (100%)	
**c.109-2A > C**	1 (+2)	0.33 (+104)	0.55(+13)	-	∆(E3p11)	∆(E3)	∆(E3p11) (85%)/∆(E3) (15%)	
**c.210A > G**	-	-	-	0.25 (+49)	-		FL (100%)	
**c.210A > C**	-	-	-	0.28(+49)	-		FL (100%)	
**c.211 + 1G > T**	0.42 (−103)	1 (−1)	-	0.47 (+47)	▼(E3q48)	∆(E3)	∆(E3) (73.3%)/▼(E3q48a) (26.7%)	
**c.211 + 2T > C**	0.32 (−104)	0.99 (−2)	-	0.59 (+46)	▼(E3q48)	∆(E3)	∆(E3) (48.1%)/▼(E3q48b) (51.9%)	

^1^ Bold-highlighted variants for which SpliceAI predictions are, in our opinion, accurate, rightly predicting the exact experimental read-out. ^2^ SpliceAI parameters were as follows (genome version hg38; score type raw; max distance 10,000 nt; Illumina’s pre-computed scores yes). Acceptor loss (AL), donor loss (DL), acceptor gain (AG), and donor gain (DG) scores (and positions) are shown. Color codes indicate scores in the 20–49 (high recall), 50–79 (recommended), and 80–100 (high precision) ranges, as per Illumina’s specifications. Scores < 20% are not shown. SpliceAI positions are annotated as (−) if upstream of the variant or (+) if downstream. Yet, the SpliceAI annotation (relative to the forward strand) becomes confusing for genes located in the antisense strand, such as *PALB2*. For that reason, in the present table, upstream (+) and downstream (−) positions are not shown as per SpliceAI but relative to *PALB2* coding strand. A minimum of two scores above the threshold are required to predict a specific aberrant outcome (e.g., for a variant damaging a donor site, an acceptor loss scoring at the right position predicts exon skipping, while a donor gain will predict use of a cryptic/de novo site). Since SpliceAI predictions for c.46A >G, c.210A >G, and c.210A >C do not fulfill the two score approach, they were considered negative (no splicing alteration) and therefore accurate.

**Table 4:** Splicing outcomes of *PALB2* variants.

Variant (HGVS) ^1^	Bioinformatics Summary (MES) ^2^	Canonical Transcript	PTC-Transcripts ^3^	In-Frame Transcripts ^4^
Wild type mgPB2_ex1–3		100%		
c.46A > G	(↓) 5′SS (5.74→ 4.05)	92.5% ± 0.1%	∆(E1q17) (7.5% ± 0.1%)	
c.48 + 1del	(−) 5′SS (5.74→ −12.45)	-	∆(E1q17) (100%)	
c.48 + 1G > C	(−) 5′SS (5.74→ −2.53)	-	∆(E1q17) (90.8% ± 0.6%)	▼(E1q9) (9.2% ± 0.6%)
c.48 + 1G > T	(−) 5′SS (5.74→ -2.76)	-	∆(E1q17) (100%)	
c.48 + 2T > G	(−) 5′SS (5.74→ −1.9)	-	∆(E1q17) (100%)	
c.48 + 5C > T	(↓) 5′SS (5.74→ 4.05)	100%		
c.49-2del	(−) 3′SS (9.28→1.16) (+) 3′SS (8.65) 6 nt downstream	-		∆(E2p6) (100%)
c.49-1del	(−) 3′SS (9.28→ −7.59) (+) 3′SS (8.76) 6 nt downstream	-		∆(E2p6) (100%)
c.49-1G > A	(−) 3′SS (9.28→0.53) (+) 3′SS (7.53) 6 nt downstream	-		∆(E2p6) (100%)
c.108 + 1_108 + 2insC	(-) 5′SS (10.86→ −4.16)	-		∆(E2) (100%)
c.108 + 5G > A	(↓) 5′SS (10.86→8.76)	100%		
c.109-2A > C	(−) 5′SS (10.06→2.02) (+)3′SS (5.47) 11 nt downstream	-	∆(E3p11) (85% ± 0.5%) ∆(E3) (15% ± 0.5%)	
c.210A > G	(↓) 5′SS (8.76→6.15)	100%		
c.210A > C	(↓) 5′SS (8.76→6.89)	100%		
c.211 + 1G > T	(↓) 3′SS (8.76→ 0.26)	-	∆(E3) (73.3% ± 0.6%)	▼(E3q48a) (26.7% ± 0.6%)
c.211 + 2T > C	(−) 3′SS (8.76→1.01)	-	∆(E3) (48.1% ± 7.4%)	▼(E3q48b) (51.9% ± 7.4%)

^1^ Bold font: No traces or <5% of the full-length transcript. ^2^ (−) site disruption; (+) New site; (↓) Reduction of MES score; Cr., Cryptic. ^3^ PTC, Premature Termination Codon; ^3,4^ Δ, loss of exonic sequences; ▼, inclusion of intronic sequences; E (exon), p (acceptor shift), q (donor shift). When necessary, the exact number of nt inserted or deleted is indicated. For example, transcript ▼(E1q9) denotes the use of an alternative donor site that is located nine nucleotides downstream of exon 1, causing the addition of 9 nt to the mature mRNA.

As above mentioned, total splicing disruptions were exclusively due to changes in the canonical AG/GT dinucleotides (Table 4). There are only a few exceptions of non-spliceogenic ±1,2 variants, basically consisting of the change of the consensus AG or GT dinucleotides into atypical splice sites, such as the GC 5′ splice sites that account for about 1% of human 5′SS [46,47]. Thus, it has been assessed that about 15–18% of + 2T > C variants, which generate an atypical GC donor site, are able to produce the full-length transcript [48], as is the case of the *PALB2* c.108 + 2T > C variant [28]. However, in this study, we have shown that c.211 + 2T > C produced just aberrant transcripts similarly to variant c.48 + 2T > C [28]. SpliceAI analysis of c.211 + 2T > C (donor loss = 0.99) correctly predicted a total impact on splicing. Curiously, we found another atypical splice-site recognition in a previous study [29]. ATM variant c.1898 + 2T > G creates an intronic GG dinucleotide that might represent an extremely rare 5′SS (~0.01% of human exons) [47]. In fact, we found that this GG 5′SS was used in 13% of minigene transcripts producing the full-length isoform [29]. Therefore, the splicing outcomes of any variant should be carefully analyzed since the generation and use of active atypical sites may rescue the production of the full-length transcript, and thus, these data may modify the clinical classification of variants. Most importantly, up to now, the use of uncommon splice sites cannot be predicted, so they can only be detected by splicing assays. In this regard, minigenes provide a substantial advantage over RNA assays in carriers since variant read-out is not mistaken with wt allele expression. Then, any residual full-length transcript produced by the variant can be tracked by the highly sensitive fluorescent fragment electrophoresis. Conversely, partial splicing outcomes producing the full-length transcript are not simply identified in patient RNA assays unless a coding heterozygous SNP was also present so that the wild-type and variant alleles can be distinguished.

Concerning the splicing output, SpliceAI produced reliable predictions for 12 out of the 16 assayed variants (Table 3). Interestingly, two false-positive variants selected on MES score (c.48 + 5C > T and c.108 + 5G > A) were ruled out by SpliceAI. MES accurately predicted splice-site disruptions or their weakening for the twelve spliceogenic variants although estimations failed in the case of four variants. By increasing the MES threshold to −30%, the specificity of the selection procedure would have considerably improved. We firmly believe that bioinformatics predictions are only useful to filter out variants and select those potentially spliceogenic, but at present, RNA assays are critical at validating a splicing effect.

### 3.3. ACMG/AMP-Based Interpretation of Variants

*PALB2* expert panel specifications of the ACMG/AMP guidelines are not yet available (https://clinicalgenome.org/, last accessed on 07 July 2022); so, as indicated in Materials and Methods, we classified 16 *PALB2* variants according to generic ACMG/AMP-based classification guidelines combined with some *PALB2* specifications previously developed by our group [28]. This approach integrates mgPALB2 readouts as observable PVS1_O/BP7_O evidence codes (Table 5). Thus, the three PTC-NMD transcripts (∆(E1q17), ∆(E3p11), and ∆(E3)) are considered a very strong evidence of pathogenicity (P_VS). Likewise, the in-frame transcript ∆(E2) deletes a key PALB2 domain (CC domain), where residues Leu17, Leu21, Leu24, Tyr28, Thr31, and Leu35 mediate important interactions in the PALB2 homodimer and/or the PALB2/BRCA1 heterodimer [28,49]. Then, ∆(E2) was deemed a very strong evidence of pathogenicity (P_VS). In addition, the in-frame isoforms ▼(E1q9) and ∆(E2p6) are predicted to disrupt critical regions for PALB2, inserting three or deleting two amino acids at the CC domain, respectively. However, in both cases, a functional impact on protein function cannot be predicted. Therefore, as we had previously pointed out [28], we think that both transcripts provide a moderate evidence of pathogenicity (P_M).

On the other hand, the contribution of ▼(E3q48) a and b (insertion of 16 new amino acids: VKSRPFTYACFIIHFP and GKSRPFTYACFIIHFP, respectively) is unclear. As we previously reported, the 16-aminoacids insertion was classified as a supporting evidence of pathogenicity (P_P) based on bioinformatics predictions (PROVEAN score of –15.84, deleterious) [28]. Finally, the FL-transcript with the missense variant c.46A > G (p.Lys16Glu) was considered a supporting benign evidence BP4 (−1) since the REVEL score (0.075) suggests no impact on protein function.

All the 16 variants are absent in the gnomAD database, so they meet the PM2 rarity code (Table 5) that we have considered a supporting evidence of pathogenicity (PM2_P; +1 point) as previously mentioned [29]. As indicated above (see Section 2.5), once we incorporate minigene readouts into the classification scheme, predictive splicing codes PVS1 (GT-AG variants) or PP3/BP4 (non GT-AG variants) are no longer taken into consideration.

Finally, we considered that some pathogenic (PS2, PM1, PM6, PP2, PP4, PP5) and benign (BS2, BP1, BP3, BP5, BP6) codes are not applicable to the classification of any of the herein described *PALB2* variants. In addition, the PM3 evidence (in trans with a pathogenic variant in a recessive disorder) was not applied to any of the variants because they were not found in Fanconi Anemia patients (based on ClinVar database, Leiden Open Variation Database, https://databases.lovd.nl/shared/genes/PALB2, accessed 8 July 2022, and literature searches).

Taking these considerations altogether, six variants were classified as likely pathogenic (+9 points of the Bayesian scale), five as VUS (+2 or +3 points), and five as likely benign (–1 or –3 points) (Table 5). Remarkably, five ± 1,2 variants (c.49-2del, c.49-1del, c.49-1G >A, c.211 + 1G >T, and c.211 + 2T >C) were catalogued as VUS because they produced the in-frame transcripts ∆(E2p6) (100% of the overall expression) or ▼(E3q48) (27%–52% of the overall expression), whose impact on PALB2 function is uncertain. Therefore, it is essential to elucidate if these transcripts retain the DNA repair activity to ascertain the pathogenicity of these five variants. Hence, the PVS1 splicing predictive evidence of ±1,2 variants may lead to their clinical misinterpretation [50].

## 4. Conclusions

We tested 16 variants at *PALB2* exons 1 to 3 by hybrid minigenes. Twelve variants impaired splicing, and eleven produced negligible levels of the mgFL-transcript. Integrating our previous results for *PALB2* [28], we analyzed a total 58 potential spliceogenic variants, 47 of which (81%) induced splicing anomalies, supporting the high sensitivity and specificity of our selection criteria as well as the efficacy of our minigene approach. By an ACMG/AMP-based strategy, a total of 29 variants were classified as pathogenic/likely pathogenic and, equally relevant, 13 variants as likely benign, whereas 16 variants were kept as VUS. Interestingly, another 56 ClinVar variants at exons 4 to 12 (accessed on 3 August 2021) would be potentially spliceogenic as per MES scores (data not shown), so in future projects, they could be assayed in our two previously reported *PALB2* minigenes: mgPALB2_ex4-6 and mgPALB2_ex5-12 [28]. Moreover, the ACMG/AMP-based guidelines provide a useful framework for the clinical interpretation of variants when splicing data are available. Finally, minigene assays allowed assessing more than 600 variants of the main breast cancer susceptibility genes up to now, demonstrating their high simplicity and robustness. Furthermore, this tool has been used to successfully assay variants at other disease genes, such as *UGT1A1* (Crigler–Najjar syndrome) [51], *CHD7* (Charge syndrome) [52], or *TRPM4* (colorectal cancer) [53], among others (http://www.ibgm.med.uva.es/servicios/servicio-de-splicing-minigenes/, accessed on 13 July 2022).

## Figures and Tables

**Table 1:** Mutagenesis primers of *PALB2* variants.

Variant	Exon/Intron	Primers (5′→3′)
c.46A > G	Ex1	CTGTGAGGAGAAGGAAGAGGTGCCGGGGGTGCGGGAAGGG
		CCCTTCCCGCACCCCCGGCACCTCTTCCTTCTCCTCACAG
c.48 + 1del	IVS1	AGCTGTGAGGAGAAGGAAAAGGGGCCGGGGGTGCGGGAAG
		CTTCCCGCACCCCCGGCCCCTTTTCCTTCTCCTCACAGCT
c.48 + 1G > C	IVS1	GCTGTGAGGAGAAGGAAAAGCTGCCGGGGGTGCGGGAAGG
		CCTTCCCGCACCCCCGGCAGCTTTTCCTTCTCCTCACAGC
c.48 + 1G > T	IVS1	TCAGCTGTGAGGAGAAGGAAAAGTTGCCGGGGGTGCGGGA
		TCCCGCACCCCCGGCAACTTTTCCTTCTCCTCACAGCTGA
c.48 + 2T > G	IVS1	CAGCTGTGAGGAGAAGGAAAAGTGCCGGGGGTGCGGGAAG
		CTTCCCGCACCCCCGGCACTTTTCCTTCTCCTCACAGCTG
c.48 + 5C > T	IVS1	GAGGAGAAGGAAAAGGTGCTGGGGGTGCGGGAAGGGCGGA
		TCCGCCCTTCCCGCACCCCCAGCACCTTTTCCTTCTCCTC
c.49-2del	IVS1	TGCCCAGTATTGTTGGTGTTTTTCTTCTTCCGTTAAAGGA
		TCCTTTAACGGAAGAAGAAAAACACCAACAATACTGGGCA
c.49-1del	IVS1	TGCCCAGTATTGTTGGTGTTTTTCTTCTTCCATTAAAGGA
		TCCTTTAATGGAAGAAGAAAAACACCAACAATACTGGGCA
c.49-1G > A	IVS1	TTCTTCCAATTAAAGGAGAAATTAGCATTCTTGAAAAGGG
		CCCTTTTCAAGAATGCTAATTTCTCCTTTAATTGGAAGAA
c.108 + 1_108 + 2insC	IVS2	CCTTCAGGCTAAGTGAATCGTATTCTCAAATTAAGGTGTT
		AACACCTTAATTTGAGAATACGATTCACTTAGCCTGAAGG
c.108 + 5G > A	IVS2	TAGCCCGCCTTCAGGTAAATGAATCGTATTCTCAAATTAA
		TTAATTTGAGAATACGATTCATTTACCTGAAGGCGGGCTA
c.109-2A > C	IVS2	TTTGTCTCCTCTCGCGTGCCCAAAGAGCTGAAAAGATTAA
		TTAATCTTTTCAGCTCTTTGGGCACGCGAGAGGAGACAAA
c.210A> G	Ex3	CCGCAGCTAAAACACTCGGGTAAATCTAGACCATTCACTT
		AAGTGAATGGTCTAGATTTACCCGAGTGTTTTAGCTGCGG
c.210A > C	Ex3	CGCAGCTAAAACACTCCGGTAAATCTAGACCATTCACTTA
		TAAGTGAATGGTCTAGATTTACCGGAGTGTTTTAGCTGCG
c.211 + 1G > T	IVS3	CCGCAGCTAAAACACTCAGTTAAATCTAGACCATTCACTT
		AAGTGAATGGTCTAGATTTAACTGAGTGTTTTAGCTGCGG
c.211 + 2T > C	IVS3	ACCGCAGCTAAAACACTCAGGCAAATCTAGACCATTCACT
		AGTGAATGGTCTAGATTTGCCTGAGTGTTTTAGCTGCGGT

**Table 5:** ACMG/AMP-based classification of 16 *PALB2* variants at exons 1 to 3.

Variants	ClinVar ^1^	ACMG-AMP ^2^ Classification	Splicing Predictive PVS1/PP3 ^3^	PVS1_O/BP7_O (mgPALB2 Readouts) ^4^	PM2 ^5^
c.46A > G (p.Lys16Glu)	B	LB (−1)	PP3	BP7_O_M (-2): 93% [BP7_O_M, FL] ^6^ + 7% [PVS1_O, ∆(E1q17)]	PM2_P (+1)
c.48 + 1del	LP	LP (+9)	PVS1	PVS1_O (+8): 100% [PVS1_O, ∆(E1q17)]	PM2_P (+1)
c.48 + 1G > C	Conflicting	LP (+9)	PVS1	PVS1_O (+8): 91% [PVS1_O, ∆(E1q17)]+ 9% [PVS1_O_M, ▼(E1q9)]	PM2_P (+1)
c.48 + 1G > T	P/LP	LP (+9)	PVS1	PVS1_O (+8): 100% [PVS1_O, ∆(E1q17)]	PM2_P (+1)
c.48 + 2T > G	LP	LP (+9)	PVS1	PVS1_O (+8): 100% [PVS1_O, ∆(E1q17)]	PM2_P (+1)
c.48 + 5C > T	Conflicting	LB (−3)	PP3	BP7_O_S (-4): 100% [BP7_O_S, FL] ^7^	PM2_P (+1)
c.49-2del	VUS	VUS (+3)	PVS1	PVS1_O_M (+2): 100% [PVS1_O_M, ∆(E2p6)]	PM2_P (+1)
c.49-1del	P	VUS (+3)	PVS1	PVS1_O_M (+2): 100% [PVS1_O_M, ∆(E2p6)]	PM2_P (+1)
c.49-1G > A	LP	VUS (+3)	PVS1	PVS1_O_M (+2): 100% [PVS1_O_M, ∆(E2p6)]	PM2_P (+1)
c.108 + 1_108 + 2insC	LP	LP (+9)	PVS1	PVS1_O (+8): 100% [PVS1_O, ∆(E2)]	PM2_P (+1)
c.108 + 5G > A	VUS	LB (−3)	PP3	BP7_O_S (-4): 100% [BP7_O_S, FL] ^7^	PM2_P (+1)
c.109-2A > C	LP	LP (+9)	PVS1	PVS1_O (+8): 85% [PVS1_O, ∆(E3p11)] + 15% [PVS1_O, ∆(E3)]	PM2_P (+1)
c.210A > G (p.Ser70=)	Conflicting	LB (−3)	PP3	BP7_O_S (-4): 100% [BP7_O_S, FL] ^8^	PM2_P (+1)
c.210A > C (p.Ser70=)	LB	LB (−3)	PP3	BP7_O_S (-4): 100% [BP7_O_S, FL] ^8^	PM2_P (+1)
c.211 + 1G > T	P/LP	VUS (+2)	PVS1	PVS1_O_P (+1): 73% [PVS1_O, ∆(E3)] + 27% [PVS1_O_P, ▼(E3q48a)]	PM2_P (+1)
c.211 + 2T > C	LP	VUS (+2)	PVS1	PVS1_O_P (+1): 48% PVS1_O, ∆(E3)] + 52% [PVS1_O_P, ▼(E3q48b)]	PM2_P (+1)

^1^ Clinical interpretation at the ClinVar database (accessed on 9 September 2022). ^2^ Point-based variant classification categories are defined as follows: pathogenic (P) ≥+10; likely pathogenic (LP) +6 to +9; variant of uncertain significance (VUS) 0 to +5; likely benign (LB) −1 to −6; and benign (B) ≤−7. ^3^ The predictive splicing codes were not taken into account in this study since they were considered redundant when splicing assay data are available. ^4^ Deconvolution of minigene readouts and assigned score according to the rules indicated in Materials and Methods. Note that transcripts representing <10% of the overall expression (e.g., ∆(E1q17) in c.46A > G) do not contribute to the final PVS1_O/BP7_O evidence strength assignment. If two transcripts representing >10% of the overall expression each support different evidence strengths, the most conservative strength is assigned (e.g., c.211 + 1G > T minigene readout ends up as PVS1_O_P even if the major signal supports PVS1_O). ^5^ Rarity code PM2 with allele frequency ≤0.01% at gnomADv2.1.1. ^6^ The FL transcript carries a missense variant r.46A >G (p.Lys16Glu) that qualifies for protein predictive evidence BP4 (multiple lines of computational evidence suggest no impact) with moderate strength (REVEL score = 0.075). Based on that, FL expression as observed in the assay qualifies for BP7_O_M. ^7^ The FL transcripts has a wt sequence that qualifies for BP7_S. ^8^ The FL transcripts carries a synonymous variant r.210A > G or r.210A > C that, once an impact on splicing has been excluded, qualifies for BP7_O.

## Data Availability

All sequencing and fragment analysis data will be available after publication at Digital. CSIC.
